# Acidic Offense: Proton-secreting Cells Orchestrate Inflammation and Sperm Damage in Epididymitis

**DOI:** 10.1093/function/zqaf044

**Published:** 2025-09-16

**Authors:** Ming Wang, Zhenyu Zhou, Sudhanshu Bhushan

**Affiliations:** Medical Research Center, The First Affiliated Hospital of Zhengzhou University, Zhengzhou University, Zhengzhou 450052, China; Medical Research Center, The First Affiliated Hospital of Zhengzhou University, Zhengzhou University, Zhengzhou 450052, China; Institute of Anatomy and Cell Biology, Unit of Reproductive Biology, Justus-Liebig-University Giessen, Giessen 35392, Germany

## A Perspective on “Proton-secreting Cells as Drivers of Inflammation and Sperm Dysfunction in LPS-induced Epididymitis”

As a critical segment of the male reproductive tract, the epididymis functions as a transitional zone for post-testicular sperm maturation and storage.^1^ This organ concurrently faces contrasting immunological demands: tolerance toward spermatozoa and defense against pathogens ascending from the urethra and vas deferens. Tightly controlled immune homeostasis is thus essential for maintaining epididymal integrity and function.^2^ Epididymitis, a common intrascrotal inflammation among young adults, typically arises when pathogenic bacteria—primarily *Escherichia coli, Staphylococcus*, and *Streptococcus* species—enter retrogradely via the vas deferens. This bacterial invasion triggers inflammation, inducing immune cell accumulation, tissue damage, fibrotic remodeling, and ultimately epididymal duct obstruction. Such structural compromise directly impairs sperm maturation, transit, and fertility.^3,4^

Crucially, specialized proton-secreting cells, resident in the epididymis, kidney, and respiratory tract, contribute substantially to immunoregulation.^5,6^ Within the epididymal epithelium, proton-secreting clear cells (CCs) are strategically positioned alongside mononuclear phagocytes (MPs) to balance immune tolerance and inflammatory responses.^1^ CCs establish an acidic luminal microenvironment through apical V-ATPase-dependent H⁺ secretion—a process indispensable for male fertility. This acidification precisely regulates pH and ionic homeostasis, creating conditions necessary for sperm maturation while providing antimicrobial protection.^7^ Analogously, renal intercalated cells mediate urinary acidification and serve as sensors of renal injury and infection. Emerging evidence positions epididymal CCs as central regulators of inflammatory signaling and mucosal immunity.^8^ Nevertheless, the specific molecular pathways and regulatory networks through which CCs coordinate immune regulation remain uncharacterized.

In a recent *Function* study, Da Silva et al. employed fluorescence-activated cell sorting to isolate CCs from proximal (initial segment/caput), middle (corpus), and distal (cauda) regions of the murine epididymis^8^This characterization of region-specific gene expression profiles utilized an intratesticular Lipopolysaccharide (LPS)/saline injection model. In saline-injected controls, proximal epididymal CCs demonstrated upregulated acid-base transport and sperm function-related genes alongside downregulated V-ATPase subunits and pro-inflammatory genes. Middle-region CCs exhibited elevated pro-inflammatory gene expression and reduced sperm maturation genes, while distal CCs showed prominent expression of sperm maturation and acid-base transport genes. Crucially, saline injection itself induced segment-specific transcriptional variation, underscoring the inherent complexity of epididymal regionalization and the sensitivity of distinct segments to retrograde flow and shear stress.

Following LPS stimulation, CCs universally downregulated sperm maturation genes while upregulating pro-inflammatory genes. The cauda region displayed the most pronounced transcriptional alterations relative to saline controls, indicating heightened susceptibility to external stimuli. Principal component analysis confirmed significant transcriptomic segregation across epididymal regions. Notably, transcriptomic changes extended to non-injection sites, revealing widespread region-specific effects. These findings align with our prior observations in a uropathogenic *Escherichia coli*-induced epididymitis model, where the distal epididymis exhibited robust pro-inflammatory gene upregulation and neutrophil/monocyte infiltration, while the caput displayed minimal transcriptional changes at day 10 post-infection.^1^ This evidence establishes fundamental regional heterogeneity in the epididymal immune landscape.

LPS stimulation further induced morphological changes in CCs. Though CC numbers remained unchanged, proximal regions developed increased apical blebbing, while distal regions showed irregular cellular architecture compared to saline controls. These membrane protrusions were apoptosis-independent. Concurrently, all regions upregulated genes governing vesicle formation, endocytosis, tight junction integrity, and actin cytoskeleton regulation. Given established roles of epididymal extracellular vesicles (EVs) in sperm maturation through protein/RNA transfer—and their documented involvement in immune responses—they hypothesize that LPS-induced CC blebs may regulate inflammation initiation and disrupt sperm maturation through EV-mediated mechanisms. This premise requires empirical validation.

As core immune sentinels, resident MPs and CCs cooperatively maintain microenvironmental homeostasis during sperm maturation through precise immunomodulation.^9,10^ The epididymal epithelium establishes its acidic luminal environment via CC activity while coordinating protein transfer to spermatozoa, thereby regulating maturation and storage homeostasis. MPs extend lumen-probing projections essential for antigen surveillance. However, LPS stimulation profoundly altered MP behavior, reducing tubulointerstitial contact frequency of CX3CR1⁺ MPs. This diminished physical interaction with CCs potentially compromises tissue homeostasis and coordinated immunity. Concurrently, dendritic cells (DCs) migrated from the caudal region to draining lymph nodes. Ly6G⁺ neutrophils infiltrated the distal interstitium, with neutrophil extracellular traps (NETs) histologically confirmed in the cauda via N-elastase positive structures—indicating NETosis-mediated antimicrobial defense. These regional responses mirror our UPEC model, confirming distinct immunological patterning: Distal regions exhibit DC migration and neutrophil recruitment, with earlier activation kinetics in the caudal segment.

Acute and chronic epididymitis frequently impair sperm count and motility. Although sperm concentration remained unchanged 24 hours post-LPS exposure, both total and progressive motility decreased significantly. This likely stems from LPS-induced dysregulation of V-ATPase subunits, luminal pH imbalance, and altered sperm maturation gene expression across segments.

Collectively, Da Silva et al. provide a comprehensive analysis of CCs in orchestrating immune responses during LPS-induced epididymitis. This work significantly advances the understanding of epididymal immunoregulation and its implications for male fertility. The study establishes that regionally positioned CCs pivotally initiate antimicrobial responses, rapidly adopting a pro-inflammatory phenotype characterized by cytokine/chemokine upregulation. Accompanying morphological changes—including apical protrusions and cellular reshaping—demonstrate functional activation during inflammation. Nevertheless, mechanisms governing CC-MP synergy and the etiology of distal CC morphological abnormalities remain uncharacterized. As research continues to unravel epididymal physiology, CCs will undoubtedly emerge as prime therapeutic targets for reproductive health management.
